# USDA Special Supplemental Nutrition Program for Women, Infants and Children (WIC) Vendor Criteria: An Examination of US Administrative Agency Variations

**DOI:** 10.3390/ijerph18073545

**Published:** 2021-03-29

**Authors:** Matthew J. Landry, Kim Phan, Jared T. McGuirt, Alek Ostrander, Lilian Ademu, Mia Seibold, Kathleen McCallops, Tara Tracy, Sheila E. Fleischhacker, Allison Karpyn

**Affiliations:** 1 Stanford Prevention Research Center, School of Medicine, Stanford University, Palo Alto, CA 94305, USA; matthewlandry@stanford.edu; 2 Harvard College, Harvard University, Cambridge, MA 02138, USA; kim_phan@college.harvard.edu; 3 Department of Nutrition, University of North Carolina Greensboro, Greensboro, NC 27412, USA; jtmcguir@uncg.edu; 4 School of Public Health, University of Michigan, Ann Arbor, MI 48109, USA; ostranda@umich.edu; 5 College of Liberal Arts and Sciences, University of North Carolina Charlotte, Charlotte, NC 28223, USA; lademu@uncc.edu; 6 Center for Research in Education & Social Policy, College of Education & Human Development, University of Delaware, Newark, DE 19716, USA; mseibold@udel.edu (M.S.); kamcca@udel.edu (K.M.); tetracy@udel.edu (T.T.); 7 Georgetown University Law Center, Washington, DC 20001, USA; sef80@georgetown.edu

**Keywords:** WIC, women, infants and children, Federal Nutrition Assistance Program, health food access, healthy in-store marketing, food policy

## Abstract

The food retail environment has been directly linked to disparities in dietary behaviors and may in part explain racial and ethnic disparities in pregnancy-related deaths. The Special Supplemental Nutrition Program for Women, Infants and Children (WIC), administered by the United States Department of Agriculture, is associated with improved healthy food and beverage access due to its requirement for minimum stock of healthy foods and beverages in WIC-eligible stores. The selection and authorization criteria used to authorize WIC vendors varies widely from state to state with little known about the specific variations. This paper reviews and summarizes the differences across 16 of these criteria enacted by 89 WIC administrative agencies: the 50 states, the District of Columbia, five US Territories, and 33 Indian Tribal Organizations. Vendor selection and authorization criteria varied across WIC agencies without any consistent pattern. The wide variations in criteria and policies raise questions about the rational for inconsistency. Some of these variations, in combination, may result in reduced access to WIC-approved foods and beverages by WIC participants. For example, minimum square footage and/or number of cash register criteria may limit vendors to larger retail operations that are not typically located in high-risk, under-resourced communities where WIC vendors are most needed. Results highlight an opportunity to convene WIC stakeholders to review variations, their rationale, and implications thereof especially as this process could result in improved policies to ensure and improve healthy food and beverage access by WIC participants. More work remains to better understand the value of state WIC vendor authorization authority, particularly in states that have provided stronger monitoring requirements. This work might also examine if and how streamlining WIC vendor criteria (or at least certain components of them) across regional areas or across the country could provide an opportunity to advance interstate commerce and promote an equitable supply of food across the food system, while ensuring the protection for local, community-oriented WIC vendors.

## 1. Introduction

The food retail environment has been directly linked to disparities in dietary behaviors and may in part explain racial and ethnic disparities in pregnancy related deaths [1,2,3]. The United States Department of Agriculture (USDA) Special Supplemental Nutrition Program for Women Infants Children (WIC) is the largest public health nutrition assistance program focused on reducing infant mortality and improving health of women who are pregnant, postpartum and/or breastfeeding, infants, and children up to age five who nutritionally are at risk and living in or near poverty [4]. The federally funded, state-operated nutrition program was authorized initially in 1972 as a two-year pilot program as part of the amended Child Nutrition Act of 1966 (P.L. 92-443) [5]. In 1975, WIC was made permanent (P.L. 94-105) and is part of the forthcoming Child Nutrition Reauthorization [5,6].

Participants are eligible for WIC if they are determined to be at nutritional risk by a health professional and garner an income at or below 185% of the US Poverty Income Guidelines, or are enrolled in Temporary Aid for Needy Families (TANF), the USDA Supplemental Nutrition Assistance Program (SNAP), Medicaid, or other means-tested programs depending on the administrative agency [7,8]. The WIC program provides supplemental food and beverages (called a nutrition prescription or the WIC food package) and as well as nutrition education, breastfeeding support, and referrals for healthcare and social services through grants provided from the USDA Food and Nutrition Service (FNS) to administrative agencies [4]. The WIC program is widely considered to be one of the most successful nutrition intervention policies [4,9]. Research has shown that participation in WIC is associated with improvements in infant and child health outcomes [10,11,12,13,14], nutrition intake and diet-related outcomes [15,16], and access to health care [17,18]. In 2019, the average number of women, infants, and children receiving WIC benefits each month was approximately 6.4 million, with an average monthly food package per person of $40.90 [19].

WIC operates through 89 WIC administrative agencies, including the 50 US states, the District of Columbia, 33 Indian Tribal Organizations (ITOs), and five US Territories [4,7]. These administrative agencies provide governance for over 1900 local agencies and 10,000 clinic sites [7]. The administrative agencies are allowed to establish criteria for vendor selection and authorization in addition to the minimum Federal requirements outlined in the Code of Federal Regulations, 7 CFR §246.12 (2020) (see Supplemental Table S1). Additional vendor selection and authorization criteria are used by the administrative agency to ensure both the lowest practicable food prices consistent with participant access, and effective management, oversight, and review of authorized vendors. Applicable administrative agencies’ vendor selection and authorization criteria are intended to ensure that vendors adhere to certain standards for cleanliness, minimum stock, WIC signage, days and hours of operation, and other similar standards that affect food access, availability, and safety for WIC participants.

The public health literature emphasizes the importance of creating equity in built environments as a framework for building health where we live, work, study, and play [20]. The food environment is one such component and has been tied to disparities in diet [21,22]. Residents in particularly high-risk, under-resourced communities can travel considerable distances to reach full-service grocery stores [23] which raises important questions about WIC retail guidelines and their scope of influence as a policy driver of the food access landscape. Such variations in access to retail food outlets, which have been tied to disparities in dietary behaviors during non-pandemic times, have intensified as a result of the Coronavirus Disease of 2019 (COVID-19) [24]. The pandemic has exacerbated food retail access concerns, including a heightened awareness of the fragility of our food system and the need for a robust local food access infrastructure [25].

In 2009, the WIC program implemented its first substantial revisions to the allowable supplemental foods and beverages since its authorization in 1972 [26]. New guidelines required increased availability of healthy foods and beverages such as whole grain breads, low-fat milks, and brown rice along with the introduction of a Cash Value Benefit to enable the purchase of fruits and vegetables (FVs). In compliance with this change, WIC vendors were required to maintain minimum stock of such products, although guidelines differ by state [27]. Research published in the last 10 years reflects considerable interest in understanding the feasibility and impacts of the 2009 WIC program changes on WIC participant diet, obesity, and food and beverage availability, as well as on breastfeeding practices [15,28,29,30,31]. Administrative vendor selection and authorization criteria and vendor management and operations policies, however, are not well understood [27,32], and to date there is no research which describes and compares criteria and policies across administrative agencies, nor any single database which houses such information, although state vendor manuals and other related program materials can be shared on the National WIC Association’s WIC Hub (thewichub.org, accessed on 26 March 2021).

The present study begins to address this gap and provides a new, foundational understanding of how administrative agency vendor selection and authorization criteria differ and, further, how these differences may contribute to differences in WIC retailer availability or usage, and to disparities in food access among income-eligible families. In documenting these differences, findings could inform federal, tribal, territorial, state, and local efforts aimed at increasing WIC participant access to vendors offering WIC thus improving community food access. Improving access to WIC foods may increase WIC participation retention as difficulty finding and shopping for WIC foods is a leading cause of early exit from the WIC program [33]. Specifically, our research sought to examine WIC vendor selection and authorization criteria across all 89 administrative agencies (i.e., 50 US states, 33 ITOs, the District of Columbia, five US Territories) in order to describe the nature and frequency of variations in policies across these geographic units.

## 2. Materials and Methods

Beginning in October 2019, a subgroup of the HER NOPREN WIC Learning Collaborative convened to develop a protocol that defined relevant data sources and variables of interest for this study. The Collaborative is a team of researchers, practitioners, and advocates committed to improving the health of women who are pregnant and lactating, as well as of infants and young children through improved research, policy and practice of the USDA WIC program. This Collaborative is a joint effort of the Healthy Eating Research (HER), a national program of the Robert Wood Johnson Foundation, and the Nutrition and Obesity Policy and Research Network (NOPREN)—a multi-disciplinary research network based out of the US Centers for Disease Control and Prevention. In addition to HER NOPREN WIC Learning Collaborative representation, the subgroup included liaisons from the National WIC Association. The subgroup identified data categories through internal dialogue and through discussion based on these criteria: (1) likely variation across states, (2) potential for influence on the local food retail landscape, and/or (3) influence on in-store product promotion or display. The data categories that were ultimately established included those that function to support both vendor selection and authorization as well as vendor management and operations. Next, definitions were established to ensure consistency in documentation. Table 1 describes data categories with operational definitions and examples pertaining to their use.

The Code of Federal Regulations applicable to WIC (7 CFR §246) was consulted; however, the regulations did not define several key topic areas for research or contained inadequate definitions for our purposes. Accordingly, we utilized the 89 administrative WIC agencies’ source documents to conduct data extraction, to the extent these documents were available. These source documents included administrative agency plans, training manuals, application documents, and vendor manuals (i.e., the documents designed to assist authorized WIC vendors and their staff in maintaining compliance with the program’s rules and regulations as they pertain to the store’s day to day WIC operations) for the most recent years, 2018–2020.

Between June and October 2020, a team of 10 research assistants worked to gather the source documents applicable to the 89 WIC-authorized administrative agencies as previously described and identified herein. For the purposes of this paper, “states” include the 50 US states and the District of Columbia. The process began with web-based searches for the source documents. Where items could not be identified on the web, administrative agency administrative staff were contacted directly via email and phone to obtain available information. Research staff persisted in contacting these administrators until: (1) they were formally denied access to materials, or (2) no return emails or calls were received after five attempts.

Ultimately all state and DC WIC vendor selection and authorization criteria (*n* = 51) were located and tabulated. Partial to complete information was available for some ITOs and US Territories (ITOs, *n* = 13; Territories, *n* = 3); see Supplemental Tables S2 and S3. A small number of ITOs (*n* = 2) were defined as ‘direct distribution’, meaning that the ITO provides WIC-approved foods directly to participants without using a vendor in a retail setting. Proof of SNAP Retailer Status was identified for one additional territory/ITO increasing sample size for only this variable to (*n* = 19). No information was available for the remaining ITOs (*n* = 18) or US Territories (*n* = 2).

After a final protocol was finalized, a team of five members undertook coding responsibilities across all administrative agencies. The team met weekly to discuss data abstractions, confusing language, and missing data. Initially team members were trained using a “test administrative agency” whereby data were extracted and compared to a gold standard sample. Team members walked through discrepancies to clarify definitions and to identify where to find needed data within administrative agency documents. During these weekly meetings, definitions were revised as needed to improve clarity and better capture content. Variables of interest were initially entered in a database as direct quotes from the source documents and were later summarized using categorical descriptors designed by the research team for each variable. For example, the categorical descriptors for “minimum number of cash registers” were: (1) At least one, (2) Three, (3) Dependent on WIC volume sales requirement, and (4) Not listed. The process allowed for the research team to initially understand the scope of variation, and then progress to design appropriate categorical options for analysis.

In order to ensure inter-rater reliability and consistency in coding, a random sample of 10 administrative agencies where coding had already been completed was selected for audit. Across the five data collectors, random combinations of two data collectors, each of whom had not before coded that agency, were assigned to re-code categorical designations; they performed this exercise independently and were blind to prior evaluator’s determinations. Results were compared to identify inconsistencies in definitional understanding. The process revealed consistent understanding of definitions with only a few improvements needed; however, it yielded new learnings for the inconsistencies in agency documentation wherein its source documents were not always aligned. As a result, the team refined the process to require that researchers first review the vendor manual for information, relying on its contents as the preferred source of information, since it was often the most complete document with regard to containing needed data. After a second round of review, it was demonstrated that the revised document utilization process, coupled with improved definitions, resulted in consistency across researcher data entries. Descriptive statistics were calculated for all variables. In order to display information visually to assess geographic and regional patterns including clustering of variables of interest, Geographic Information System (GIS) (ArcGIS Pro 2.3, Environmental Systems Research Institute (ESRI), Redlands, CA, USA) was used to visualize the spatial distribution of coded variables at the state level. Data were joined to a US state boundary shapefile and colored symbology was generated based on quantitative values.

## 3. Results

### 3.1. Store Specific Vendor Seletion and Authorization Policies

**Figure 1:**
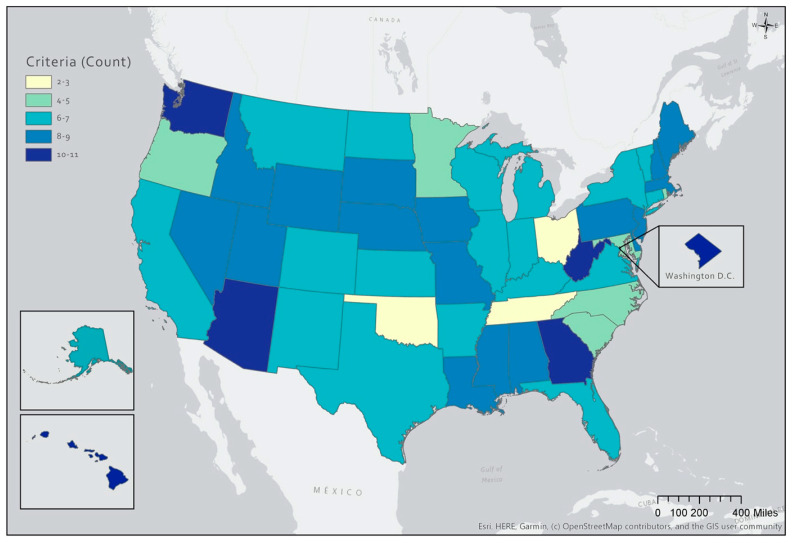
Total Number of Vendor Selection and Authorization Criteria Adopted by State Agency-Authorized WIC Vendors, 2018–2020.

A state-by-state sum of the 13 store vendor selection and authorization criteria examined are presented in Supplemental Table S4. Number of criteria ranged from 2 to 11 with a mean of 7. Oklahoma had the fewest criteria (*n* = 2) and Washington, DC had the most criteria (*n* = 11). As depicted in Figure 1, there was no clear regional pattern across the states. Findings related to the 13 store-specific vendor criteria are summarized in Table 2.

#### 3.1.1. Store Hours of Operation

Eight distinct parameters for store hours of operation (h) (including no requirement) were identified (Table 2). Vendor criteria for store h of operation varied from a specific number of h per day (6, 8, 9 or 10 h), to blocks of time per day (two 4-h blocks of time), to total number of h per week. The most frequent requirements for number of h across states were eight h per day (39.2%, *n* = 20) and 10 h per day (15.7%, *n* = 8). Overall, the second largest number of states had no specific requirement for h of operation (21.6%, *n* = 11). The balance of the states that have requirements are split between a set number of h per day (66.7%, *n* = 34), a set number of h per week (9.8%, *n* = 5), and one state that requires h dependent on store type (2%, *n* = 1).

#### 3.1.2. Store Days of Operation

Three distinct store days of operation requirements (including no requirement) were identified (Table 2). The majority of states (68.8%, *n* = 35) require vendors to be open for business six days per week, although many (19.6%, *n* = 10) have no specified requirement for days of operation. A smaller number (11.8%, *n* = 6) require their vendors to be open five days a week. Guidelines in US Territories and ITOs most often require stores to remain open eight hours per day (61.1%, *n* = 11) and six days per week (66.7%, *n* = 12).

#### 3.1.3. Minimum Number of Registers

In most states (68.6%, *n* = 35) vendors are required to have at least one register, while other states do not specify a requirement for a minimum number of registers (19.6%, *n* = 10). A few states (Delaware, Mississippi, and Washington, DC) require three registers (5.9%, *n* = 3), or have register specifications that are dependent on vendor sales (5.9%, *n* = 3). Territories and ITOs most often had no specified requirement for the minimum number of registers (72.2%, *n* = 13).

#### 3.1.4. Minimum Square Footage of WIC Retail Store

The majority of states had no specified requirement for store size (80.4%, *n* = 41). States that had specified requirements were primarily concentrated in the southeast and northeast US. Where specified, parameters varied; these distinct criteria (in square feet, sq ft) included minimums of: 1000 sq ft (*n* = 4); 2000 sq ft (*n* = 1); 3000 sq ft (*n* = 2); 9000 sq ft (*n* = 1); and, 10,000 square ft (*n* = 2). Despite being some of the smallest areas by land in the US, Delaware and the District of Columbia had the highest requirement (≥10,000 square ft) for minimum square footage. The majority of territories and ITOs (88.9%, *n* = 16) had no specified requirement for minimum square footage.

#### 3.1.5. Full-Service Grocery Only Criteria

Less than half the states (39.2%, *n* = 20) require WIC vendors to be a full-service grocery store, while slightly more than half of the states (52.9%, *n* = 27) allow full-service grocery stores as well as other store types. Half of the territories and ITOs (50%, *n* = 9) limit vendors to only full-service grocery stores, while some (22.2%, *n* = 4) allow a range of stores which can include full-service stores.

#### 3.1.6. A50 or WIC Only Stores

Almost three-quarters of the states (74.5%, *n* = 38) prohibit A50 or WIC-only stores, while 11 states (21.6%) allow these: Alabama, Arizona, California, Florida, Georgia, Indiana, Louisiana, New Mexico, New York, Oklahoma, and Texas. The remaining states do not clearly specify whether A50 or WIC-only vendors are eligible for approval. Territories and ITOs had a nearly equal split between allowing A50 or WIC-only stores (18.4%, *n* = 7) and prohibiting them (21.1%, *n* = 8).

#### 3.1.7. Pharmacy as Redemption Site

Almost three-quarters (72.5%, *n* = 37) of the states allow pharmacies to sell some type of WIC-approved foods although they may be limited to infant formula only or medical foods. Conversely, about 1 in 6 states (15.7%) expressly prohibit pharmacies to become WIC-approved food vendors. Most territories and ITOs (61.1%, *n* = 11) do not have a specified requirement regarding pharmacies; where criteria are set, they are split between allowing and not allowing pharmacies.

#### 3.1.8. Required to Be an Established Store

Six states (11.8%) require vendors to be in business for at least a year or more while the remaining states (88.3%, *n* = 45) have no requirement or did not specify a requirement related to the amount of time a store must be open before it is eligible to become a vendor. Territories and ITOs most commonly had no requirement (50.0%, *n* = 9) or did not specify (27.8%, *n* = 5) a length of time for which a store must be established before it is eligible to become a vendor.

#### 3.1.9. Clean/Orderly Store

Just over half of states (54.9%, *n* = 28) specify that vendors must maintain a clean/orderly store whereas 23 states had no such specified requirement. Vendors in most territories and ITOs (66.7%, *n* = 12) must adhere to a clean/orderly store requirement.

#### 3.1.10. “Good Standing” Store Requirement

Over 70 percent of states (72.5%, *n* = 37) clearly describe in criteria that stores are required to be in “good standing,” or that vendors were compliant with current permitting regulations. Similarly, the majority of territories and ITOs (77.8%, *n* = 14) require that the vendor be in “good standing”.

#### 3.1.11. Grocery Class Permit Requirement

Slightly more than half of states (51.1%, *n* = 26) require vendors to hold a grocery class permit (i.e., to adhere to applicable state standards for business operation, public health, and/or food sales). The practice is less common in territories and ITOs (21.1%, *n* = 8) where no such guidance was indicated.

#### 3.1.12. Proof of SNAP Retailer Status

All states require vendors to have proof of SNAP retailer eligibility status, consistent with Federal regulations. The vast majority of territories and ITOs (84.2%, *n* = 16) clearly designate the need for proof of SNAP status as an eligibility requirement. Of note, USDA FNS issues permits to qualified retailers to accept SNAP benefits, monitors SNAP stores to ensure they follow SNAP program rules, and withdraws or disqualifies SNAP stores who have broken rules or no longer qualify to accept SNAP benefits.

#### 3.1.13. WIC Volume Sales Requirement

Less than one-third of states (29.5%, *n* = 15) require WIC vendors to meet volume sales requirements (i.e., percentage or dollar amount of WIC sales over an established time period). Nearly all of territories and ITOs (83.3%, *n* = 15) have no requirement for their vendors to meet any volume of WIC sales. One territory or ITO (2.6%) required their vendors to meet certain volume of WIC sales.

### 3.2. Vendor Management and Operations Policies

**Figure 2:**
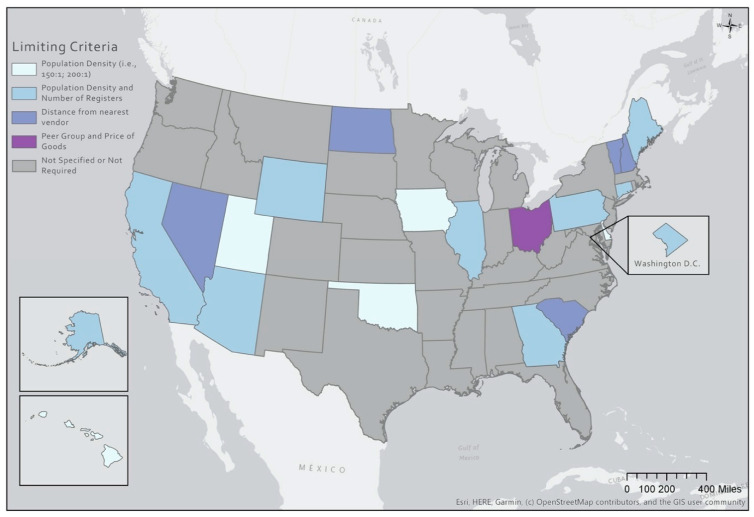
Limiting Criteria Adopted by State Agency-Authorized WIC Vendors, 2018–2020.

Findings related to vendor management and operations policies are summarized in Table 3 and Table 4, and in Figure 2.

#### 3.2.1. Limiting Criteria

Over half of states do not specify limiting criteria (58.8%, *n* = 30), while nearly one-fifth (19.6%, *n* = 10) require vendor assignment based on population density and number of registers. The remainder of states use limiting criteria that is based on vendor to participant ratios, peer groups and prices of WIC goods, and distance between approved vendors. Territories and ITOs used population density as limiting criteria the most frequently (44.4%, *n* = 8). The remaining territories and ITOs either used distance from the nearest approved vendor (22.2%, *n* = 4) as limiting criteria or had no specified limiting criteria (22.2%, *n* = 4). A map of the criteria is provided in Figure 2.

#### 3.2.2. Parameters for Shelf Talkers or Shelf Tags to Label WIC Products, Including Criteria for Talkers/Tags

Over 75 percent (76.5%, *n* = 39) of the states allow all WIC-approved foods to be labeled as a WIC-approved product on store shelves, while approximately two-thirds of the states (68.7%, *n* = 35) establish criteria for these labels which, in most cases, encompasses specific parameters such as label content or size (Table 3). Similarly, most territories and ITOs (61.1%, *n* = 11) allow shelf talkers or shelf tags to label all WIC products.

#### 3.2.3. WIC Product Grouping Criteria

A majority of the states and D.C. do not have guidelines outlining whether vendors are allowed to group WIC products in the store (72.5%, *n* = 37). In contrast, ten states specifically did not allow WIC vendors to group WIC products, while four states permitted the practice. The majority of territories and ITOs (83.3%, *n* = 15) had no specified requirement about grouping WIC and non-WIC products together.

#### 3.2.4. Peer Group Criteria

The findings related to peer group criteria, as a vendor management and operation policy, are presented separately and as follows due to its complexity. Eight distinct peer grouping classification options (Table 4) were identified, including:

1. Store type
2. Size
3. Geography
4. Cash registers
5. Ownership type
6. Food basket price
7. Transportation access
8. Amount of Sales

Among these, the most common in states are groupings by store type (73.5%, *n* = 36), geography (required, unless an exemption is received; 77.6%, *n* = 38), and/or number of cash registers (44.9%, *n* = 22). Of particular note is the geography classification, which is a nationally required peer group criterion unless an exemption is allowed by USDA. However, a number of states (*n* = 10) did not list specific geographical criteria; one state noted its exemption from this criterion. Trends are similar among territories and ITOs.

## 4. Discussion

Considerable revisions were made to the WIC program as part of the Healthy, Hunger-Free Kids Act of 2010 (P.L. 111–296), including a shift from paper benefits to WIC Electronic Benefit Transfer (EBT) (81 FR 10433) (2016). Historically WIC vouchers were utilized via paper coupons requiring clients to use their entire monthly benefit at the same time in the same store; however, in nearly all states this is no longer the case [34]. New WIC EBT processes enable clients to utilize benefits to purchase eligible products across the month, so WIC clients no longer risk sacrificing benefits should a store not have a certain item in stock [35]. The contextual shift to EBT therefore calls to question the rationale for some of the foundation guidance states have relied on, and gives rise to new opportunities to revisit WIC vendor selection and authorization criteria. Such a shift in operational process, opens new doors to coordination between WIC and SNAP including the potential to resolve capacity issues at state WIC agencies to monitor vendor compliance. Further, there may be new opportunities to strengthen WIC engagement in the local retail community.

This study is the first to our knowledge to compile and examine a database of WIC vendor selection and authorization criteria and operations and management policies established by the 89 administrative agencies in the US. Our findings reflect the complexity of obtaining and examining these parameters, particularly for territories and ITOs where vendor criteria and policies were often not available. After many attempts, no vendor selection and authorization criteria could be obtained for 20 of the 38 territories or ITOs. Such effort suggests that a central, searchable system to maintain state, tribe, territory, and local agency information is needed, or at minimum, the information should be housed on a central website, or in a consistent location on administrative agency websites. The benefits of this system would facilitate understanding of individual and administrative agencies’ vendor selection and authorization framework as well as comparison between frameworks, as not all administrative agencies post this information online or are even able to distribute it upon request via email. This knowledge may be beneficial to policymakers, research and advocacy organizations, product distributors, and of course, WIC vendors and participants themselves. Future multi-disciplinary work could examine the most critical administrative agency variations needed to allow for contextual and cultural adaptations and to stimulate innovations, while also ensuring the free flow of commerce between states is not obstructed (US Constitution, Article 1, Section 8, Clause 3). This could include simulation modeling to identify the most effective and efficient policies for WIC vendors and product distributors, often working through regional food distribution centers and complying with varying administrative agencies’ operations and management policies [36,37].

Our research lends new insight into several vendor selection and authorization criteria, that, in combination, result in a policy mechanism which may unintentionally limit the quality of the food environment in low- and moderate-income areas. The characteristics of stores used by administrative agencies to determine eligibility, such as minimum square footage, number of registers (beyond federal requirements), or full-service status vary without any consistent pattern. If a large percentage of WIC participants live in lower income neighborhoods, they may have more barriers to accessing WIC vendors in the states with increased or more stringent requirements. Furthermore, the accessibility of retailers which offer a variety of healthy products in communities may have implications for other food access efforts, such as additional monies for fruit and vegetable benefits, voucher and/or incentive programs. A study by Zenk et al. (2014) measured the impact of the fruit and vegetable voucher added to the WIC-approved food packages on fruit and vegetable prices found that WIC participants’ purchasing power differed depending on the type and neighborhood of the WIC vendor used [38]. Indeed, for many WIC products, variation in cost is less important to the consumer, because it is redeemed by item and not by cost. However, this issue can be significant for fruit and vegetables which WIC participants purchase by a set dollar amount rather than by number of items.

Prior research involving WIC vendors is often in the context of either adherence to minimum stocking criteria (including the 2009 changes to these criteria) or examination of the overall healthfulness of products available at the store [32,39,40,41,42]. Findings from these studies generally show little variability in store offerings among larger grocery stores and big box WIC retail vendors. However, medium and small store WIC vendors, when compared to non-WIC authorized competitors, are more likely to offer more healthful products. Results from this examination suggest that smaller WIC vendors and distributors may be disproportionally impacted by WIC vendor eligibility criteria. Vendor monitoring requirements represent a sizable burden to states, which often have to fulfill requirements with very limited state agency staff. As a consequence, states may limit vendor eligibility to narrow requirements to fewer stores. One other area of consideration, which was not undertaken in this study, is the role that state specific infant formula and jarred baby food specifications may have on limiting vendor participation.

Limiting the landscape of WIC vendors may have substantial impacts on communities. Research has shown that increasing the number of WIC authorized vendors results in increased availability of healthy foods and beverages, benefiting both WIC-participants and non-WIC participants [43]. Research has also demonstrated that authorizing smaller stores as WIC redemption sites can improve the healthfulness of products not just in these small stores, but also in neighboring non-WIC authorized competitor stores as well [44]. Authorization to accept WIC benefits has advantages for retailers. Research has found that becoming a vendor led to an increases in both sales of healthy, WIC-eligible foods and in average weekly dollar sales, in comparison to similar stores that were not authorized as a WIC vendor [45]. Discount variety retailers provide an opportunity to provide healthy foods and beverages to low-income rural areas and urban food deserts [45,46]. These small-format stores sell an array of food, household, and other miscellaneous products at reduced prices. However, these retailers often operate a regional distribution model, and meeting WIC vendor criteria that varies from region to region or state to state can be challenging. More research remains to better understand the value of state WIC vendor authorization authority, particularly in states that have provided stronger monitoring requirements. This work might also examine if and how streamlining WIC vendor criteria (or at least certain components of them) across regional areas or across the country could provide an opportunity to advance interstate commerce and promote an equitable supply of food across the food system, while ensuring the protection for local, community-oriented WIC vendors.

In-store marketing approaches for healthy foods and beverages are well established and viable mechanisms to increase their consumption [47]. Strategies aligned with the “4 P’s” of marketing (product, price, promotion, placement) have demonstrated effectiveness at increasing product sales, both alone and in combination. The present study found that 75% of states allow stores to include WIC shelf tags on all eligible items, and slightly fewer (69%) have established parameters to do so. Results raise questions about the rationale for limiting labeling in the 25% of states that do so, and also open doors for new opportunities to understand, and potentially update, established labeling criteria, given the evolution of research on healthy in-store marketing practices [22,48,49,50]. For example, working with retailers, including thought leaders such as large, big box retailers, to establish a national WIC-approved labeling program, along with revised state guidance, could result in more efficient benefit access and use for both customers who now redeem benefits via EBT across multiple shopping trips and the retailers that sell WIC-approved products. More work remains to explore how improvements in nationwide uniformity of WIC labeling policies might better align with the general trend for federal preemption in promulgating nutrition labeling as put forth by the Nutrition Labeling and Education Act (NLEA) (P.L. 101-535).

## 5. Conclusions

WIC is designed to influence lifetime nutrition and health behaviors in a targeted, high-risk population; however, the wide range of vendor selection and authorization criteria raises questions about the rationale for the inconsistency found by the current research, particularly given the importance of local access to healthy foods and beverages. One of WIC’s goals is to reduce disproportionate health outcomes across social and economic groups by providing access to WIC-approved foods to participating populations. With the forthcoming Child Nutrition Reauthorization, findings suggest an opportunity for Congress to hold inquiries on opportunities to further improve the WIC program. First, there is a need for a common database to house all administrative agencies’ WIC plan information, including vendor selection and authorization criteria, so that variations in requirements across states, ITOs, and territories are able to be analyzed and considered alongside other user data. Given the variations across administrative agencies in these criteria, and the lack of apparent pattern guiding a rationale for the variations, there is an opportunity to convene WIC stakeholders to review variations, their rationale, and implications thereof, since this process could result in improved policies to ensure and improve healthy food access by WIC participants. Our results suggest too that there is an opportunity to revisit the potential for WIC product labeling among the 25% of states that do not expand labeling to all WIC products. Finally, findings provide an opportunity in the 75% of states that allow labeling of all WIC products to examine, as a next step, their characteristics such as their content, color, imaging, dimensions, and frequency of re-positioning to ensure that they align with healthy in-store marketing best practices.

## Figures and Tables

**Table 1:** Data Categories with Operational Definitions and Exemplars from Administrative Agency WIC Vendor Selection and Authorization Criteria and Vendor Management and Operations Policies, 2018–2020.

Data Category	Operational Definition	State Agency (Selected)	Administrative Agency Guidance Example
**Vendor Selection and Authorization Criteria**			
Store Hours of Operation	The required hours and days of the week, or minimum total number of hours throughout the week that eligible WIC retail stores must be open.	Vermont	The store must be in a permanent location and be open a minimum of 8 hours per day, 6 days per week.
Store Days of Operation	The required days of the week that eligible WIC retail stores must be open.	California	All WIC vendors must have a fixed location and remain open 8 hours a day, six days a week, including at least four hours during core business hours of 9AM to 5 PM.
Minimum Number of Registers	The minimum number of Point-of-Sale systems a store must have to be eligible as a WIC vendor.	Washington	Have at least 1 electronic cash registers capable of producing receipts that include the store name, food product name and description, quantity sold, price of each item, total actual purchase price, and the date of sale.
Minimum Square Footage of WIC Retailer Store	The minimum square footage the state requires that eligible WIC retail stores maintain for food sales and storage.	Kansas	Vendors must provide foods from stationary locations, have a minimum food sales area of 2000 square feet or more, and be accessible to clients with disabilities. Military Commissaries are considered retail grocery stores.
		Mississippi	WIC Vendors must have a minimum of 9000 square feet of continuous retail space allocated solely for food products.
Full-Service Grocery Criteria	May include the term “full-service,” or more general descriptions of “grocery stores” or “retail grocery.”	Alabama	The store must be a business whose primary purpose is to be a retail grocer. Retail grocery does not include the following: gas stations, specialty stores, liquor stores, home delivery groceries, bait shops, etc.
		Arkansas	A full-service grocery store stocking MUST HAVE at a minimum, all of the following food groups: canned, fresh, and frozen fruits and vegetables, fresh and frozen meats and poultry (pre-packaged luncheon meats and deli meats do not qualify as meeting that requirement), canned fish, dairy products such as milk, eggs, and cheese, cereals, breadstuffs, canned and dry beans, pasta and infant foods and infant formula in order to qualify.
		Rhode Island	The grocer applicant must stock a variety of staple foods for sale including fresh, frozen and/or canned fruits and vegetables, fresh, frozen and/or canned meats, daisy products, and grain products such as bread, rice and pasta and a minimum inventory and supply of WIC-Approved Foods at competitive prices.
A50 or WIC-only Stores	A50 stores derive more than 50% of their annual revenue from WIC sales (hence Above 50 or A50); WIC-only stores serve only WIC participants.	Nebraska	Store sales must meet the following criteria: No more than 20% of the retailer’s gross annual total sales may be from alcoholic beverages. No more than 50% of the retailer’s gross annual retail food sales (actual or anticipated) may be from the WIC program. Stores that only stock and sell WIC approved foods, also known as “WIC Only Stores”, are not eligible for authorization as an approved Nebraska WIC Retailer. Store applicants may be required to submit supporting documentation to verify sales information.
		Louisiana	Vendors that derive more than 50 percent of their annual food sales revenue from WIC FIs, and new vendor applicants expected to meet this criterion under guidelines approved by FNS. A50 Vendors are subject to payment limitations that ensure that the prices of A50 Vendors do not result in higher total food costs if Program participants transact their food instruments at A50 Vendors rather than at non-A50 (“regular”) Vendors.
Pharmacy Allowed as Vendor	Specifies if pharmacies or drug stores may be authorized as WIC vendors in order to sell infant formula or medical foods (typically).	Kentucky	A Drug Store or Pharmacy is only authorized to provide exempt formula or WIC Eligible Nutritionals. No other foods or formulas may be redeemed by a drug store/pharmacy. A drug store must be able to supply exempt formula or WIC Eligible Nutritionals within forty-eight (48) hours of verbal request. Have a recognized pharmacy section in a stationary location that is a separate and distinct area.
		Alaska	Pharmacies may be authorized to provide medical or specialized infant formulas to WIC participants.
Established Store (≥1 year)	Specifies if the state requires WIC vendor applicants to be open for a specified amount of time, prior to becoming an eligible authorized WIC vendor.	Arizona	The Department shall verify that the Applicant’s store is a full line grocery store and a viable business that has been open for at least one (1) year.
		Missouri	Vendor applicant must have been in business in the current location for at least a year.
Clean and Orderly Store	Parameters detailing how an establishment must be maintained in a clean, orderly, and safe condition, with no current sanctions for violations of local health code ordinances, and/or compliance with applicable Federal, State and local health protection laws and ordinances.	Louisiana	Maintain the establishment in a clean, orderly and safe condition, with no current sanctions for violations of the Louisiana state Sanitary Code (LAC 51), the International Plumbing Code as amended by the Louisiana State Uniform Construction Code Council (LAC 17:I.111), or local health code ordinances.
		Delaware	Have a valid public health permit and maintain the store in a clean and sanitary condition per the State of Delaware Food Code.
“Good Standing” Store Requirement	Specifies if vendors must adhere to local and current permitting regulations and must not be in violation of SNAP retail standards.	Nevada	The vendor must be in good standing and cannot be, or has been in the preceding two years, disqualified or suspended from the Food Stamp Program/SNAP, or been assessed a Food Stamp Program/SNAP civil money penalty for hardship if the disqualification period that would otherwise have been imposed has not expired.
		California	Vendor or Vendor’s ownership must maintain their business entity in good standing with the jurisdiction of incorporation or organization. The business entity must not be suspended, canceled, dissolved, or under any other status that renders the business entity unable to legally operate or otherwise engage in business transactions.
Grocery Class Permit Requirement	Specifies if WIC vendors are required to possess a state-issued grocery sales license or permit, or the equivalent, in their state.	Maine	Possession of a valid Food Establishment License from the Maine Department of Agriculture, Food and Rural Resources (or its equivalent from another state) or registration as a pharmacy through the Maine Board of Pharmacy (or its equivalent from another state).
		Minnesota	Must possess Minnesota Retail Food Handlers License issued by the Minnesota Dept of Agriculture and City or County Grocer’s License or operating license if your city or county issues those licenses.
Proof of SNAP Retailer Status	Specifies in guidance that WIC vendors are required to prove their authorization as a SNAP retailer prior to authorization.	New York	Any changes to SNAP authorization must be reported; In addition, violations of WIC Program regulations can cause you to lose your authorization in the Supplemental Nutrition Assistance Program (SNAP).
		Utah	Prior to WIC authorization, the vendor applicant must have applied for SNAP authorization and must provide their FNS/SNAP Number as part of the WIC Vendor Agreement Application.
WIC Volume Sales Requirement	The minimum dollar amount of WIC sales, during a specific time period, as evidence of a WIC vendor’s foundation in selling required WIC-approved foods.	Michigan	A Vendor that transacts less than $2400 per quarter of WIC EBT [Electronic Benefit Transfer] transactions will be considered as low volume and will be treated as lack of demand for a particular store. A Vendor that falls below this quarterly volume threshold may be subject to Contract termination; and disqualification from WIC Program participation.
		New Hampshire	The State agency may deny authorization if, for retail food stores only, the vendor’s monthly average volume of WIC business over the most recent 12 months is less than $200.00 and another authorized vendor is located within 2 miles, unless inadequate participant access is determined.
**Vendor Management and Operations Policies**			
Limiting Criteria	Parameters (e.g., distance between WIC stores) determining allowable WIC vendor locations, creating adequate access points to WIC-approved foods that can be adequately managed by state agencies.	Georgia	The primary method for regulating the number of authorized vendors is through the use of a vendor-to- participant ratio. The vendor-to-participant ratios are determined prior to each application/authorization period. For vendor authorization, exceptions to the vendor-to-participant ratio conclusions may be considered under the following circumstances: The need to ensure that each food instrument issuance clinic site in the state has an authorized vendor within a 10-mile radius; The need to provide adequate service to participants in a population center of at least ten (10) individuals who have no access to an authorized vendor within a 10-mile radius of the population center.
		California	The State shall set criteria to limit the number of retail vendors in the WIC system. The State will use the following vendor-limiting criteria: (1) prices charged are within peer group pricing limits; (2) ability of CDPH [California Department of Public Health] to ensure WIC foods are provided via compliance monitoring; (3) the adequacy of WIC foods stocked on store shelves; and (4) past vendor compliance with both WIC and CalFresh vendor laws.
Shelf labels/tags/talkers	Labels used in the store that show WIC identifying information (e.g., “WIC-approved food, logo, state agency name”), are defined and allowed by state agencies to create clear messaging regarding WIC-approved foods.	Arkansas	All vendors are required to mark the appropriate approved food items with shelf tags issued by the Arkansas WIC Program. For food categories that require the purchase of the “least expensive brand at time of purchase” tag ONLY the least expensive approved brand available in each variety in each container size. These food categories are: milk (refrigerated, dry, canned; regular, lactose-free, and acidophilus), cheese, eggs, canned beans, and juice. In the Arkansas Approved Food list, these categories list this requirement in bold print at the beginning of each section if it applies.
		Idaho	The vendor may choose to use WIC shelf tags provided by the State or create their own with prior approval. The vendor is responsible for ensuring that WIC shelf tags are properly placed to correctly identify food items listed on the current Idaho Food List. If the vendor chooses to use shelf tags in a food category, shelf tags must be placed on all WIC approved foods in that category. Shelf tag placement should be checked regularly.
		Colorado	Retailers may use shelf tags (i.e., shelf labels, flags, talkers, channel strips or clings) indicating an item is WIC eligible under the following conditions throughout the WIC agreement period. The tags must be placed at the exact spot(s) that contain the WIC approved item(s) indicated. The retailer shall be responsible that food items tagged are WIC approved. Retailers are responsible for the placement of shelf tags. Retailers who wish to develop and use shelf tags must obtain written permission from COWIC [Colorado WIC] by submitting a copy or sample of the final version for approval prior to use. WIC tags/labels are not permitted to be put on individual item containers; labels created by manufacturers stating WIC approval are not permitted. Retailers can decide which food categories in the store to use the shelf tags.
Peer Group System Criteria	State agency-established system that groups vendors with similar characteristics, one of which is geographical in nature, as a means to contain costs.	Connecticut	Peer group means a category of vendors that are assigned based on population density in the ZIP code area of the store and the number of checkout lanes or cash registers in the store.
		Oregon	Peer groups are based on the following criteria: geographic location, store model (single store, small chain, large chain, and pharmacy), and for single stores only, number of registers.

**Table 2:** Store-Specific Selection and Authorization Criteria for Administrative Agency-Authorized WIC Vendors, 2018–2020.

WIC Vendor Selection and Authorization Criteria	Number (%) of States and District of Columbia (*n* = 51)	Number (%) of Territories and ITOs, (*n* = 18) ^a^
**Store Hours of Operation**		
6 h per day	1 (2.0)	0 (0)
8 h per day	20 (39.2)	11 (61.1)
9 h per day	2 (3.9)	1 (5.6)
10 h per day	8 (15.7)	0 (0)
Two 4-h blocks of time	3 (5.9)	1 (5.6)
40–50 h/week	5 (9.8)	0 (0)
Varied h based on store type	1 (2.0)	0 (0)
Not specified	11 (21.6)	3 (16.7)
Direct Distribution ^b^	NA	2 (11.1)
**Store Days of Operation**		
5 days a week	6 (11.8)	2 (11.1)
6 days a week	35 (68.6)	12 (66.7)
Not specified	10 (19.6)	2 (11.1)
Direct Distribution	NA	2 (11.1)
**Minimum Number of Registers**		
At least one	35 (68.6)	3 (16.7)
Three	3 (5.9)	0 (0)
Dependent on amount of sales	3 (5.9)	0 (0)
Not specified	10 (19.6)	13 (72.2)
Direct Distribution	NA	2 (11.1)
**Minimum Square Footage of WIC Retailer Store**		
1000–3000	7 (13.7)	0 (0)
9000	1 (2.0)	0 (0)
10,000	2 (3.9)	0 (0)
Not specified/no requirement	41 (80.4)	16 (88.9)
Direct Distribution	NA	2 (11.1)
**Full-Service Grocery Criteria**		
Allows Range of Stores	27 (52.9)	4 (22.2)
Only Allows Full-Service	20 (39.2)	9 (50.0)
Not Specified	4 (7.8)	3 (16.7)
Direct Distribution	NA	2 (11.1)
**A50 or WIC-Only Stores Allowed**		
Yes, Allowed	11 (21.6)	7 (38.9)
No, Not Allowed	38 (74.5)	8 (44.4)
Not Specified	2 (3.9)	1 (5.6)
Direct Distribution	NA	2 (11.1)
**Pharmacy Allowed as Vendor**		
Yes, Allowed	37 (72.5)	3 (16.7)
No, Not Allowed	8 (15.7)	2 (11.1)
Not Specified	6 (11.8)	11 (61.1)
Direct Distribution	NA	2 (11.1)
**Established Store (≥1 year)**		
Yes, Required	6 (11.8)	2 (11.1)
No, Not Required	3 (5.9)	9 (50.0)
Not Specified	42 (82.4)	5 (27.8)
Direct Distribution	NA	2 (11.1)
**Clean and Orderly Store**		
Yes, Required	28 (54.9)	12 (66.7)
No, Not Required	0 (0)	0 (0)
Not Specified	23 (45.1)	4 (22.2)
Direct Distribution	NA	2 (11.1)
**“Good Standing” Store Requirement**		
Yes, Required	37 (72.5)	14 (77.8)
No, Not Required	6 (11.8)	1 (5.6)
Not Specified	8 (15.7)	1 (5.6)
Direct Distribution	NA	2 (11.1)
**Grocery Class Permit Requirement**		
Yes, Required	26 (51.1)	0 (0)
No, Not Required	20 (39.2)	8 (44.4)
Not Specified	5 (9.8)	8 (44.4)
Direct Distribution	NA	2 (11.1)
**Proof of SNAP Retailer Status**		
Yes, Required	50 (98.0)	16 (84.2)
No, Not Required	0 (0)	1 (5.3)
Not Specified	1 (1.9)	0 (0)
Direct Distribution	NA	2 (10.5)
**WIC Volume Sales Requirement**		
Yes, Required	15 (29.5)	1 (5.6)
No, Not Required	36 (70.6)	15 (83.3)
Direct Distribution	NA	2 (11.1)

Abbreviations: ITOs, Indian Tribal Organizations; SNAP, Supplemental Nutrition Assistance Program; A50, Above 50. ^a^ Proof of SNAP Retailer Status was identified for one additional territory/ITO increasing sample size for only this variable to *n* = 19. ^b^ Direct distribution refers to ITOs that provide WIC-approved foods directly to participants without using a vendor in a retail setting.

**Table 3:** Vendor Management and Operations Criteria for Administrative Agency-Authorized WIC Retailers, 2018–2020.

WIC Vendor Selection and Authorization Criteria	Number (%) of States and District of Columbia (*n* = 51)	Number (%) of Territories and ITOs, (*n* = 18)
**Limiting Criteria**		
Population Density (i.e., 150:1; 200:1)	5 (9.8)	8 (44.4)
Population Density and Number of Registers	10 (19.6)	0 (0)
Distance (from nearest vendor, a radius: 1 mile, 2 miles or driving distance, 5 miles; or, only in location where needed)	5 (9.8)	4 (22.2)
Peer Group and Price of Goods	1 (2.0)	0 (0)
Not specified	30 (58.8)	4 (22.2)
Direct Distribution	NA	2 (11.1)
In-Store WIC-Approved Labeling		
**Allows Shelf Labels, Tags, or Talkers to Identify WIC Products**		
Yes, All Products	39 (76.5)	11 (61.1)
Yes, Only Lowest Price Item	2 (3.9)	0 (0)
Yes, Other Non-Price Based Criteria Used	6 (11.8)	1 (5.6)
No	1 (2.0)	0 (0)
Not specified	3 (5.9)	4 (22.2)
Direct Distribution	NA	2 (11.1)
**Have Established Criteria for Shelf Labels, Tags, or Talkers Not Provided/Designed by the State agency/Territory/ITO**		
Established Criteria	35 (68.7)	3 (16.7)
No Established Criteria	0 (0)	0 (0)
Not Specified	16 (31.4)	13 (72.2)
Direct Distribution	NA	2 (11.1)
**Allows WIC and Non-WIC Products to be Grouped Together**		
Yes, Allowed	3 (5.9)	1 (5.6)
Yes, Experimental/Trial Period	1 (2.0)	0 (0)
No, Not Allowed	10 (19.6)	0 (0)
Not Specified	37 (72.5)	15 (83.3)
Direct Distribution	NA	2 (11.1)

Abbreviations: ITOs, Indian Tribal Organizations.

**Table 4:** Vendor Peer Group System Criteria for Administrative Agency-Authorized WIC Retailers, 2018–2020.

	Store Type	Store Size	Geography	Number of Cash Registers	Ownership Type	Food Basket Price	Transportation Access	Amount of Sales	Exempt
Number of States Using Criteria (%) ^a^	36 (73.5)	2 (4.1)	38 (77.6)	22 (44.9)	2 (4.1)	1 (2.0)	0 (0)	10 (20.4)	0 (0)
Number of Territories/ITOs Using Criteria (%) ^b^	6 (66.7)	2 (22.2)	5 (55.6)	2 (22.2)	0 (0)	0 (0)	1 (11.1)	0 (0)	1 (11.1)

Note: States and ITOs frequently use multiple criteria for grouping vendors into peer groups. ^a^ *n* = 49, for 2 states, peer groups are required but no standards were specified, ^b^ *n* = 9, for 7 Territories/ITOs, peer groups are required but no standards were specified; for 2 Territories/ITOs, direct distribution of WIC benefits is used; and for 20 Territories/ITOs, multiple contact were made but no information was received/available.

## Data Availability

The data that support the findings of this study are available on request from the corresponding author (A.K.).

## Supplementary Materials

The following are available online at https://www.mdpi.com/article/10.3390/ijerph18073545/s1, Table S1: Minimum Federal Requirements for State Agency-enacted WIC Vendor Selection and Authorization Criteria, 2018–2020; Table S2: U.S. Territories Administratively Operating WIC Programs, 2018–2020; Table S3: Indian Tribal Organizations (ITOs) Administratively Operating WIC Programs, 2018–2020; Table S4: State-by-State Numbers of WIC Vendor Selection and Authorization Criteria Adopted by State Agencies, 2018–2020.
