# Supplementary material for: USDA Special Supplemental Nutrition Program for Women, Infants and Children (WIC) Vendor Criteria: An Examination of US Administrative Agency Variations
Source: Int J Environ Res Public Health. 2021 Mar 29;18(7):3545. doi: 10.3390/ijerph18073545 (PMC8037245; doi:10.3390/ijerph18073545)
Supplement: Supplementary file 1 [file ijerph-18-03545-s001.pdf]

Special Supplemental Nutrition Program for Women, Infants and Children (WIC) Vendor Criteria: An Examination of US Administrative Agency Variations

Supplemental Tables

Supplemental Table 1: Minimum Federal Requirements for Administrative Agency-enacted WIC Vendor Selection and Authorization Criteria, 2018–2020 [1]

Requirement Category	Requirement Summary
Minimum variety and quantity of supplemental foods[2]	A vendor must stock two different fruits, two different vegetables, and at least one whole grain cereal.
Business integrity	A vendor may not be authorized if it has been convicted of an activity indicating lack of business integrity.
Current SNAP disqualification	A vendor may not be currently disqualified from SNAP.
Provision of incentive items	An above-50 percent vendor may not be authorized if it provides incentives on prohibited items.
Competitive price	Vendors are required to participate in established peer groups that create competitive pricing and allowable reimbursable levels, and that also consider participants' geographic access.

[1] Adapted from Code of Federal Regulations, 7 CFR § 246.12(g)(3) and (g)(4) (2020).
[2] "Supplemental Foods" used in the Code of Federal Regulations; this paper uses "WIC-approved foods" for clarity.

Special Supplemental Nutrition Program for Women, Infants and Children (WIC) Vendor Criteria: An Examination of US Administrative Agency Variations

Supplemental Tables

Supplemental Table 2: U.S. Territories Administratively Operating WIC Programs, 2018–2020

U.S. Territories (n = 5)	WIC Vendor Documents Available for Extraction
American Samoa	No
Guam	Yes
Northern Mariana Islands (Commonwealth of)	Yes
Puerto Rico	Yes
U.S. Virgin Islands	No

Special Supplemental Nutrition Program for Women, Infants and Children (WIC) Vendor Criteria: An Examination of US Administrative Agency Variations

Supplemental Tables

Supplemental Table 3: Indian Tribal Organizations (ITOs) Administratively Operating WIC Programs, 2018–2020

U.S. ITOs (*n* = 33)	State in Which ITO is Located	WIC Vendor Documents Available for Extraction
Acoma, Canoncito, & Laguna	New Mexico	Yes
Cherokee Nation	Oklahoma	No
Cheyenne River Sioux	South Dakota	No
Chickasaw Nation	Oklahoma	No
Choctaw Indians	Mississippi	No
Choctaw Nation	Oklahoma	Yes
Citizen Potawatomi Nation	Oklahoma	Yes
Eastern Cherokee	North Carolina	No
Eight Northern Pueblos	New Mexico	No
Five Sandoval Pueblos	New Mexico	No
Indian Township	Maine	No
Inter-Tribal Council	Arizona	Yes
Inter-Tribal Council	Nevada	Yes
Inter-Tribal Council	Oklahoma	Yes
Isleta Pueblo	New Mexico	Yes
Muscogee Creek Nation	Oklahoma	Yes
Navajo Nation	Arizona	No
Northern Arapahoe	Wyoming	No
Omaha Sioux	Nebraska	No
Osage Tribal Council	Oklahoma	Yes
Otoe-Missouria Tribe	Oklahoma	Yes
Pleasant Point	Maine	Yes
Rosebud Sioux	South Dakota	No
San Felipe Pueblo	New Mexico	Yes[a]
Santee Sioux	Nebraska	No
Santo Domingo Tribe	New Mexico	Yes[a]
Shoshone Tribe	Wyoming	No
Standing Rock Sioux Tribe	North Dakota	No
Three Affiliated Tribes	North Dakota	Yes
Ute Mountain Ute Tribe	Colorado	No
Wichita, Caddo, and Delaware Tribes	Oklahoma	Yes
Winnebago Tribe	Nebraska	No
Zuni Pueblo	New Mexico	No

[a] ITOs were defined as 'direct distribution' meaning that the ITO provides WIC foods directly to participants without using a vendor in a retail setting.

Special Supplemental Nutrition Program for Women, Infants and Children (WIC) Vendor Criteria: An Examination of US Administrative Agency Variations

Supplemental Tables

Supplemental Table 4: State-by-State Numbers of WIC Vendor Selection and Authorization Criteria Adopted by State Agencies, 2018–2020

State	Number of Vendor Selection and Authorization Criteria	State	Number of Vendor Selection and Authorization Criteria
Oklahoma	2	Texas	7
Ohio	3	Vermont	7
Tennessee	3	Wisconsin	7
North Carolina	4	Delaware	8
South Carolina	4	Iowa	8
Maryland	5	Louisiana	8
Minnesota	5	Maine	8
Oregon	5	Massachusetts	8
Rhode Island	5	Mississippi	8
Arkansas	6	Missouri	8
Colorado	6	Nevada	8
Indiana	6	New Hampshire	8
Kentucky	6	Pennsylvania	8
New Mexico	6	South Dakota	8
North Dakota	6	Wyoming	8
Virginia	6	Alabama	9
Alaska	7	Idaho	9
California	7	Nebraska	9
Connecticut	7	New Jersey	9
Florida	7	Utah	9
Illinois	7	Arizona	10
Kansas	7	Georgia	10
Michigan	7	Hawaii	10
Montana	7	Washington	10
New York	7	West Virginia	10
		Washington, DC	11
